# Efficacy of 1-α-Hydroxycholecalciferol Supplementation in Young Broiler Feed Suggests Reducing Calcium Levels at Grower Phase

**DOI:** 10.3389/fvets.2020.00245

**Published:** 2020-06-10

**Authors:** Matthew F. Warren, Thien C. Vu, Ondulla T. Toomer, Juan David Fernandez, Kimberly A. Livingston

**Affiliations:** ^1^Prestage Department of Poultry Science, North Carolina State University, Raleigh, NC, United States; ^2^United States Department of Agriculture, Agricultural Research Service, Raleigh, NC, United States; ^3^Premex, Durham, NC, United States; ^4^Elanco Animal Health, Greenfield, IN, United States

**Keywords:** 1-α-hydroxycholecalciferol, vitamin D_3_, calcium, broiler, blood chemistry, sodium phosphate cotransporter type IIb, calbindin d28k, 25-hydroxycholecalciferol

## Abstract

Increasing biopotency of cholecalciferol (D_3_) from vitamin sources is essential in the poultry industry to meet nutritional demands and counter stressors. D_3_ exhibits hormonal traits and is responsible for calcium (Ca) absorption. 1-α-Hydroxycholecalciferol (1α) is a synthetic form of D_3_ that has equal efficacy and is cheaper to synthesize than 1,25-dihydroxycholecalciferol (active form of D_3_), on broilers. However, 1α bypasses a critical regulatory point, the kidney, and may consequently lead to toxicity levels of Ca via Ca absorption. This study examined 1α supplementation in broiler diets with different Ca inclusion levels to determine if 1α at higher Ca levels caused Ca toxicity at starter and grower phases with Ross 708 male broiler chicks. In Experiment 1 (1–15 days of age), chicks were assigned to one of 10 treatment starter diets with five levels of Ca inclusion (0.80, 0.95, 1.10, 1.25, and 1.40%) with or without 1α supplementation (5 μg 1α/kg in feed) and eight replicate cages per treatment. In Experiment 2, chicks were fed common starter diet until 16 days of age, and then they were assigned to one of eight treatment diets with four levels of Ca inclusion (0.54, 0.76, 0.98, or 1.20%) with or without 1α supplementation (5 μg 1α/kg in feed). At the end of both experiments, blood was collected from broilers to determine blood chemistry, including concentrations of vitamin D metabolites. Intestinal tissues were also collected to examine gene expression. In Experiment 1, broilers not fed 1α exhibited a quadratic effect in ionized blood Ca (iCa) as dietary Ca inclusion levels increased; 1α-fed broilers displayed an increase in iCa as Ca inclusion levels increased (*p* = 0.0002). For Experiment 2, 1α-fed broilers displayed a decrease in 25-hydroxycholecalciferol plasma concentration as dietary Ca inclusion levels increased (*p* = 0.035); also, increasing Ca inclusion in diets increased expression of duodenal sodium phosphate cotransporter type II b (NPTIIb, *p* = 0.03). Our findings imply that inclusion of 1α was beneficial because 1α enhanced Ca absorption during the starter phase; however, to avoid potential Ca toxicity or antagonism while using 1α during the grower phase, there should be consideration with reducing dietary Ca levels.

## Introduction

Vitamin sources with improved bioefficacy are essential in the poultry industry to accommodate nutritional demands of rapidly growing broilers. D_3_ is necessary for accommodating fast growth of broilers by increasing absorption of calcium (Ca) and its deposition into the bones (1, 2). The biopotency of a nutrient can be enhanced by utilizing synthetic forms of the nutrient (3), increasing bioavailability to accommodate a greater response if the nutrient's metabolic effect is dose dependent (4), or adding supplemental enzymes to increase efficacy of the nutrient of interest (5). Supplemental enzymes can be costly, and their effectiveness can vary based on nutrient load and feed processing (6). Synthetic forms of a nutrient could have unintended effects and require further testing (7, 8), but they are a viable economical solution for the poultry industry (9).

**Figure 1:**
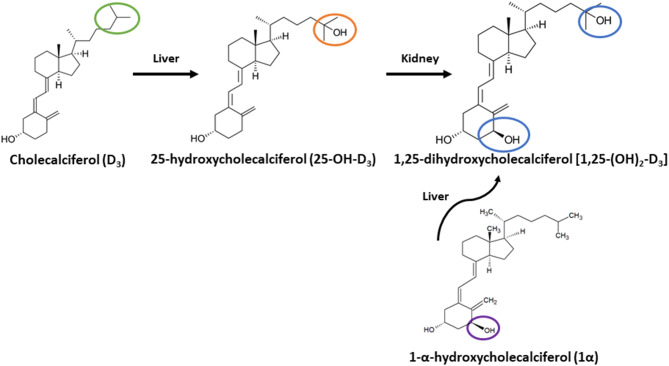
Metabolic pathway of cholecalciferol (vitamin D_3_) and 1-alpha-hydroxycholecalciferol (1α) to 1,25-dihydroxycholecalciferol (1,25-(OH)_2_-D_3_). Vitamin D_3_ is converted to 25-hydroxycholecalciferol (25-OH-D_3_) in liver, then 25-OH-D_3_ is converted to 1,25-(OH)_2_ D_3_ in Kidney. 1α travels to liver to be converted to 1,25-(OH)_2_-D_3_. Green circle highlights C-25 of D3; orange circle highlights C-25 with hydroxyl group for 25-OH-D_3_; blue circles denote C-1 and C-25 hydroxyl groups of 1,25-(OH)_2_-D_3_; purple circle highlights C-1 hydroxyl group of 1α.

Previous studies reported inclusion of 1-α-hydroxycholecalciferol (1α), a synthetic analog of vitamin D_3_, improved Ca absorption in growing broiler chicks over D_3_ alone and showed 1α having equal efficacy to 1,25-dihydroxycholecalciferol [1,25-(OH)_2_-D_3_] (10, 11). 1α is cheaper to synthesize and supply in diets compared to 1,25-(OH)_2_-D_3_ (3, 10). 1α's structure is similar to 1,25-(OH)_2_-D_3_, but only the 1-alpha carbon is hydroxylated instead of both the 1-alpha and 25-carbon. 1α has greater biopotency over D_3_ because it is quickly hydroxylated in the liver to its active form, 1,25-(OH)_2_-D_3_ (12), and consequently bypasses the hydroxylation step occurring in the kidney. In contrast, D_3_ requires two hydroxylation steps, first in the liver to form 25-hydroxycholecalciferol (25-OH-D_3_), which is further hydroxylated in the kidney to 1,25-(OH)_2_-D_3_ (Figure 1). However, 1α bypasses the critical regulatory hydroxylation by 1α-hydroxylase in the kidney (10, 13), and 25-OH-D_3_ levels might significantly increase to result in excessive Ca absorption leading to hypercalcemia (14). Although levels of ionized blood Ca (iCa) toxicity in broilers is not established, Hurwitz et al. (15) fed fast-growing chicks diets ranging from 0.4 to 2.0% Ca with 0.7% *P* diets and observed a weight loss in the fast-growing chicks.

Ca has an important relationship with phosphorus (*P*), because, together, they comprise a major part of bone structure (16). In the form of limestone or calcium carbonate, Ca is an inexpensive ingredient and is used as a carrier for many other feed ingredients including mineral premixes and drugs (17). Ca's counterpart, *P*, is one of the more expensive feed ingredients, which limits the amount incorporated into diets. This relationship can result in varying Ca: *P* ratios from 1:1 to 2.6:1 in weight (2). Studies have reported that increasing dietary Ca levels reduced incidence of tibial dyschondroplasia and therefore improved animal health, welfare, and economic value (2, 18, 19). Also, an elevated dietary Ca: *P* ratio of 2.6:1 does not appear to negatively affect tibial growth plate morphology at 2 weeks of age (20).

Although studies have been done on 1α and how it influences vitamin D status in broilers (3, 21), no studies to date have examined how 1α affects Ca absorption and vitamin D status when broilers are fed differing levels of dietary Ca. This study explores how 1α impacts vitamin D status in broiler chickens when they are fed diets with different levels of Ca in starter and grower phases.

## Materials and Methods

Two experiments were conducted to analyze effects of 1α supplementation and increasing levels of Ca inclusion on blood chemistry of starter and grower phases of broilers. All animal protocols (# 014-113 for Experiment 1 and # 17-125-A for Experiment 2) were approved by the Institutional Animal Care and Use Committee at North Carolina State University.

### Experiment 1: 1α Supplementation at Starter Phase

#### Birds and Housing

Four hundred and eighty 1-day-old Ross 708 chicks were hatched at North Carolina State University's Chicken Education Unit in Raleigh, NC. Chicks were housed in Petersime battery cages with six birds per cage and eight replicate cages per treatment. The experimental design was a completely randomized design with or without 1α supplementation (alpha D_3_, Premex, Antioquia, Colombia) at 5 μg/kg of feed [1α dose based on Snow et al. (22), because of its effectiveness] and five levels of Ca inclusion which were added on top of basal diet (Table 1) (23). For this study, broilers from dietary treatments with 1α are noted as D_3_ + 1α; broilers not fed 1α are noted as D_3_. Ca inclusion levels were 0.80, 0.95, 1.10, 1.25, and 1.40% with 0.50% available *P* in all diets, and birds were fed *ad-libitum*. The lighting program was set for 23:1 L: D hours for the first 7 days, and the last 8 days of the experiment was set to 17:7. Room temperature was set to be adjusted daily to ensure thermoneutral temperatures as birds grew. Blood was collected at 15 days from two birds per cage via brachial wing vein. At 17 days, all birds were culled. A total of 16 plasma samples per treatment, D_3_ and D_3_ + 1α, from broilers given diets at 0.95% Ca had their plasma sent (no pooling) to Heartland Assays (Ames, IA) for analysis of vitamin D metabolites by LC–MS/MS.

**Table 1:** Ingredient composition and calculated nutrient content of starter basal diet (1–17 days of age) for Ross-708 broilers [From (23)].

**Ingredient name**	**%**	**Nutrient**	**%**
Corn	53.25	Dry matter	88.55
Soybean meal, 46% CP	30.94	Moisture	11.45
Corn gluten meal	5.00	Crude protein	22.71
Poultry fat	0.00	Calcium	0.30
Soybean oil	4.20	Total phosphorous	0.38
Celite	1.00	Non-phytate phosphorous	0.18
Filler (Limestone + CaHPO_4_ + Sand)	3.15	Phytate phosphorous	0.24
Salt (NaCl)	0.29	Total methionine	0.67
DL-Methionine, 99%	0.30	Total cysteine	0.36
Sodium bicarbonate	0.31	Total lysine	1.39
Dicalcium phosphate (CaHPO_4_)^a^	0.27	Total tryptophan	0.25
Mineral premix^b^	0.20	Total threonine	0.98
Limestone Cerne Pure Cal 12–40^c^	0.17	Total isoleucine	0.94
L-Lysine-HCl, 78.8%	0.38	Total valine	1.05
Choline chloride, 60% choline	0.18	Total leucine	2.13
L-Threonine, 98%	0.15	Total arginine	1.40
Selenium premix	0.05	Total sulfur amino acids	1.04
Vitamin premix^d^	0.10	Total glycine	0.88
Anticoccidial^e^	0.05	Sodium	0.22
Natuphos E®^f^	0.01	Potassium	0.85
Total	100.00	Chloride	0.29
		Digestible lysine	1.28
		Digestible methionine	0.63
		Digestible cysteine	0.31
		Digestible total sulfur amino acids	0.94
		Digestible threonine	0.85
		Digestible tryptophan	0.22
		Digestible isoleucine	0.84
		Digestible leucine	2.04
		Digestible valine	0.95
		Digestible arginine	1.30
		Metabolizable Energy, kcal/kg	3,000
		Dietary electrolyte balance, mEq/100 g	254

a *Dicalcium phosphate contains 19.79% calcium, 17.91% phosphorus, and 17.73% available phosphorus*.

b *Trace minerals provided per kg of premix: 60 g manganese (Mn SO^4^); 60 g zinc (ZnSO^4^); 40 g iron (FeSO4); 5 g copper (CuSO^4^); 1.25 g iodine [Ca(IO^3^)^2^]*.

c *Limestone (Cerne Pure Cal 12-4) contains 39.467% calcium*.

d *Vitamins provided per kg of premix: 13,227,513 IU vitamin A; 3,968,253 IU vitamin D^3^; 66,137 IU vitamin E; 39.6 mg vitamin B12; 13,227 mg riboflavin; 110,229 mg niacin; 22,045 mg d-pantothenic acid; 3,968 mg menadione; 2,204 mg folic acid; 7,936 mg vitamin B6; 3,968 mg thiamine; 253.5 mg biotin*.

e *Coban® 90 (Monensin), Elanco Animal Health, Greenfield, IN, at 500 g/ton in the starter and grower diets*.

f *Natuphos E® (500 FTU/kg, 50 g/ton FTU)*.

### Experiment 2: 1α Supplementation at Grower Phase

#### Birds and Housing

Nine hundred and sixty Ross 708 chicks were hatched at North Carolina State University's Chicken Education Unit in Raleigh, NC. Chicks were housed in 40 floor pens with 24 birds per pen with five replicates per treatment. All chicks were fed a common starter diet (Tables 2, 3) (23) until 17 days of age. It should be noted that all starter diets for these chicks had 1α supplementation (5 μg/kg of feed). Like Experiment 1, 1α used in this experiment was alpha D_3_ from Premex (Antioquia, Colombia). Like the prior experiment, broilers from dietary treatments with 1α are noted as D_3_ + 1α; broilers not fed 1α are noted as D_3_. At 17 days of age, birds were switched to grower diet and assigned to one of eight treatment groups with four levels of Ca inclusion (added on top of basal diet; 0.54, 0.76, 0.98, or 1.20% of diet) and with or without 1α supplementation (5 μg/kg of feed) with five replicate pens per treatment. All diets contained 0.50% available *P*. At 35 days of age, blood was collected from two birds per pen and euthanized, and duodenal and jejunal tissues were collected. Duodenal tissue was washed with saline and stored in RNAlater at −20°C. Jejunal tissue was washed with saline and stored in 4% formalin for histology. Plasma from each treatment was pooled using four birds per housing row into one pooled sample, for a total of three pooled reps per treatment and sent to Heartland Assays (Ames, IA) for analysis of vitamin D metabolites.

**Table 2:** Ingredient composition of starter diet and grower basal diets for Ross-708 male broilers [From (23)].

**Ingredient name**	**Starter (1–16 d)**	**Grower (17–35 d)**
	**(%)**	
Corn	55.84	57.59
Soybean meal, 46% CP	31.70	26.89
Corn gluten meal	4.90	5.00
Soybean oil	3.12	4.85
Dicalcium phosphate (CaHPO_4_)^a^	1.37	0.31
Limestone Cerne Pure Cal 12–40^b^	1.06	0.01
Sodium bicarbonate	0.36	0.23
L-Lysine-HCl, 78.8%	0.36	0.31
DL-Methionine, 99%	0.29	0.25
Salt (NaCl)	0.28	0.28
Mineral premix^c^	0.20	0.20
Choline chloride, 60%	0.18	0.18
L-threonine	0.13	0.10
Vitamin premix^d^	0.10	0.10
Anticoccidial^e^	0.05	0.05
Selenium premix	0.05	0.05
Sand	0.01	3.60
Celite	-	1.00
Limestone dicalcium base	-	2.60
1α(OH)D_3_	0.00125	-
Natuphos E^®^^f^	0.005	0.005
Total	100.00	100.00

a *Dicalcium phosphate contains 19.79% calcium, 17.9091% phosphorus, and 17.73% available phosphorus (99%)*.

b *Limestone (Cerne Pure Cal 12-4) contains 39.01% calcium*.

c *Trace minerals provided per kg of premix: 60 g manganese (Mn SO^4^); 60 g zinc (ZnSO^4^); 40 g iron (FeSO4); 5 g copper (CuSO^4^); 1.25 g iodine [Ca(IO^3^)^2^]*.

d *Vitamins provided per kg of premix: 13,227,513 IU vitamin A; 3,968,253 IU vitamin D^3^; 66,137 IU vitamin E; 39.6 mg vitamin B12; 13,227 mg riboflavin; 110,229 mg niacin; 22,045 mg d-pantothenic acid; 3,968 mg menadione; 2,204 mg folic acid; 7,936 mg vitamin B6; 3,968 mg thiamine; 253.5 mg biotin*.

e *Coban® 90 (Monensin), Elanco Animal Health, Greenfield, IN, at 500 g/ton in the starter and grower diets*.

f *Natuphos E® (500 FTU/kg, 50 g/ton FTU)*.

**Table 3:** Nutritional content of basal starter and grower diets for Ross-708 male broilers [From (23)].

**Nutrient**	**Starter (1–16 d)**	**Grower (17–35 d)**
	**(%)**	
Crude protein	23.14	20.94
Calcium	0.87	0.24
Total phosphorous	0.58	0.36
Non-phytate phosphorous	0.38	0.18
Phytate phosphorous	0.24	0.22
Total methionine	0.65	0.59
Total cysteine	0.38	0.35
Total lysine	1.41	1.23
Total tryptophan	0.27	0.24
Total threonine	0.97	0.86
Total isoleucine	0.97	0.87
Total valine	1.09	0.98
Total leucine	2.15	2.00
Total arginine	1.43	1.27
Total sulfur amino acids	1.03	0.93
Total glycine	0.90	0.81
Digestible lysine	1.28	1.12
Digestible methionine	0.63	0.56
Digestible cysteine	0.32	0.29
Digestible total sulfur amino acids	0.94	0.85
Digestible threonine	0.85	0.75
Digestible tryptophan	0.23	0.21
Digestible, isoleucine	0.88	0.79
Digestible leucine	1.99	1.85
Digestible valine	0.96	0.87
Digestible arginine	1.33	1.18
Sodium	0.23	0.19
Potassium	0.88	0.781
Chloride	0.28	0.275
Metabolizable energy, kcal/kg	3,000.00	3,100.00
Dietary electrolyte balance, mEq/kg	267.53	227.10

### Blood Collection and Blood Chemistry

Blood was collected into BD Vacutainer lithium heparin tubes (Franklin Lakes, NJ) from two birds per cage (Experiment 1) or pen (Experiment 2). Blood chemistry parameters and iCa were determined using an i-STAT™ blood analyzer (Abaxis, Union City, CA) using CG8+ cartridges (Abaxis, Union City, CA), and the remaining blood was spun down, and plasma was collected and stored at −80°C to determine vitamin D metabolite levels. All plasma samples were sent to Heartland Assays (Ames, IA) for measuring D_3_, 25-OH-D_3_, and 24,25-dihydroxycholecalciferol [24,25-(OH)_2_-D_3_, the inactive form of D_3_] by LC–MS/MS.

### RNA Extraction and qPCR

Total mRNA was extracted from duodenal tissue using Qiagen's RNeasy Mini Kit (Germantown, MD). Extracted RNA was diluted and normalized to ~200 ng/μl and reverse transcribed to complementary DNA (cDNA) using Applied Biosystems' High-Capacity cDNA Reverse Transcription Kit (Thermo Fisher Scientific, Waltham, MA) and protocol to make a 20-μl working solution. Cycling procedure for reverse transcription started with 25°C for 10 min, 37°C for 120 min, and 85°C for 5 min and then held at 5°C indefinitely until storage or use.

Genes expressed for qPCR were vitamin D receptor (VDR) mucin 2 (MUC2), calbindin D28k (CALB), sodium phosphate cotransporter type IIb (NPTIIb), and glyceraldehyde (GAPDH) as a housekeeping gene (Table 4). qPCR was conducted using PowerUP SYBR Master Mix (Life Technologies, Grand Island, NY) using the Applied Biosystems protocol to make a 20-μl working solution and using the Applied Biosystems StepOnePlus Real-Time PCR System (Carlsbad, CA). Cycling procedure started with 95°C for 10 min and then 40 cycles of 95°C for 15 s for denaturing and 15 s at 60°C for annealing. All samples were run in triplicates.

**Table 4:** Primer sequences for quantitative real-time PCR (qPCR).

**Gene**	**Orientation**	**Primer sequence (5^**′**^−3^**′**^)**	**Size (bp)**	**Accession #**
VDR	Forward	TGCCTCCAGTCTGGCATCTC	297	NM_205098.1
	Reverse	GGTGATTTTGCAGTCCCCGT		
MUC2	Forward	GTGTGCCCTGATGTCACAGA	246	XM_015286749.1
	Reverse	GGCCTGAGCCTTGGTACATT		
CALB	Forward	TTGCCGACGGAGGAGAATTT	261	NM_205513.1
	Reverse	GGCCAGTTCAGTAAGCTCCA		
NPTIIb	Forward	AACTGGCTTGCTGTGTTTGC	423	NM_204474.2
	Reverse	GATGGCAAGATCAGGCAGGT		
GAPDH	Forward	TGTTGTTGACCTGACCTGCC	291	NM_204305.1
	Reverse	CTGGCTCACTCCTTGGATGC		

### Histology

Light microscopy (40 × magnification) was used for morphometric analysis of histological serial sections of jejuna prepared using standard hematoxylin and eosin staining to examine if dietary treatments affected gut morphology. Villus height, crypt depth, and villus width were measured using image analyzer AmScope version 3.7 (Irvine, CA). Ten measurements for villus surface area (μm^2^) and villus height/crypt depth were made per experimental unit (bird).

### Data Analysis

All data are reported as mean ± standard error of the mean. Statistical analyses were conducted using general linear model using the following model:

$$\begin{array}{l} Y_{\text{ijk}}=\mu +\beta_{1}{\left(\text{Ca}\right)}_{\text{i}}+\beta_{2}{\left(\text{Vit}.\text{D}\right)}_{\text{j}}+\beta_{3}{\left(\text{Ca}\times \text{Vit}.\text{D}\right)}_{\text{ij}}+\;\varepsilon_{\text{ijk}} \end{array}$$

where Y_ijk_ is the individual observation; μ is the experimental mean; Ca is the Ca inclusion effect of the *i*th level; Vit. D is the effect of 1α supplementation of the *j*th level; and ε_ijk_ is the error. βs are the slopes for each predictor. The interaction term of Ca × Vit. D is also included in the model. This model was used to compare differences in blood chemistry concentrations, vitamin D metabolite plasma concentrations, and relative gene expression using SAS 9.4®. The Tukey–Kramer test was used for multiple comparisons for differences between and within treatment groups for blood chemistry, vitamin D metabolites, and mRNA relative expression. All mRNA relative expressions were normalized using 2^−ΔΔ*CT*^ (24) with GAPDH as a housekeeping gene control. Statistical significance was established at *p* < 0.05, and statistical trends were noted when 0.05 < *p* ≤ 0.10.

## Results

### Experiment 1: Starter Diet

#### 1α Efficacy Dependent on Dietary Calcium Inclusion Levels With iCa Concentration

**Figure 2:**
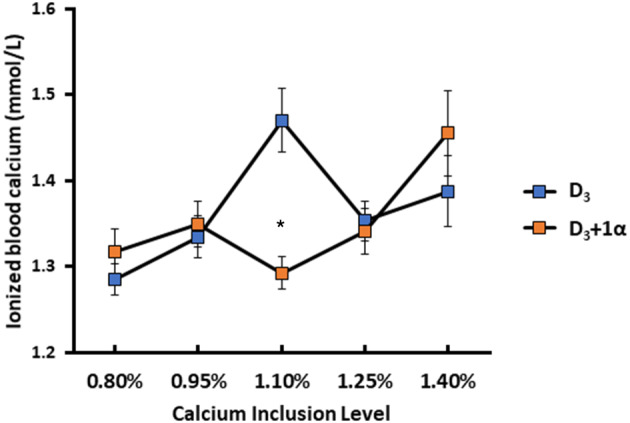
Ionized blood calcium of 15 d broiler chickens fed different levels of calcium with or without 1-alpha hydroxycholecalciferol supplementation (D_3_ + 1α; D_3_, respectively). Line graphs show means ± standard error means (*n* = 8). Interaction effect observed between calcium inclusion and 1α supplementation [General linear model (GLM), *p < 0.05].

In terms of blood chemistry, only iCa exhibited any differences between 0.80% and 1.10% Ca inclusion (Figure 2). For D_3_ + 1α broilers, iCa increased as dietary Ca increased (1.46 mmol/L iCa at 1.40% Ca inclusion). Broilers from D_3_ treatments exhibited a quadratic effect with increasing iCa from 1.29 mmol/L to a peak of 1.47 mmol/L when dietary Ca increased from 0.80 to 1.10% Ca inclusion. Once dietary Ca went over 1.10%, iCa decreased (*p* = 0.006).

#### 1α Supplementation Did Not Affect Plasma Vitamin D_3_ Levels at 0.95% Ca Inclusion

At 0.95% Ca inclusion, 1α supplementation did not affect plasma concentrations of 24,25-(OH)_2_-D_3_ or 25-OH-D_3_. A statistical trend was observed with D_3_ + 1α broilers having a higher D_3_ concentration compared to D_3_ broilers (data not shown; *p* = 0.07).

### Experiment 2: Grower Diet

#### Specific Blood Chemistry Parameters Are Affected by Ca Inclusion or 1α Supplementation

**Figure 3:**
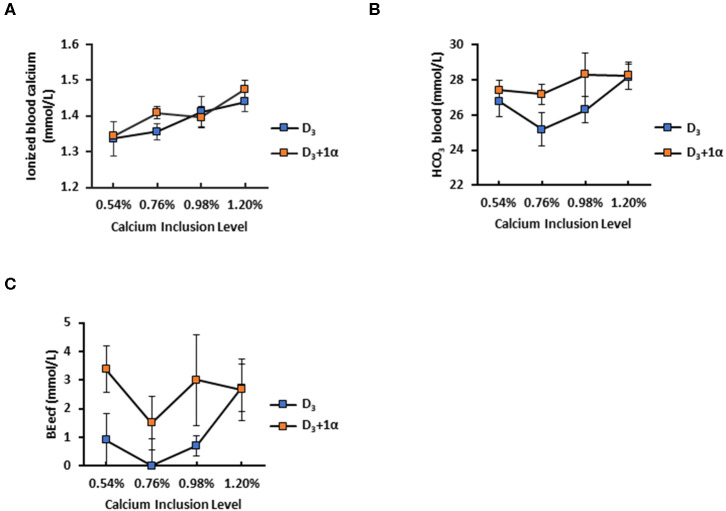
Selected blood chemistry of 35 d broiler chickens fed different levels of calcium with or without 1-alpha-hydroxycholecalciferol supplementation (D_3_ + 1α; D_3_, respectively). **(A)** Ionized blood calcium **(B)** Base excess of extracellular fluid (BEecf) **(C)** Blood bicarbonale (HCO_3_). Line graphs show means ± standard error means (*n* = 5). Calcium inclusion effect observed for ionized blood calcium; 1α supplementation effect with BEect [General linear models (GLM), *p* < 0.05].

1α had no effect on 35-days broilers' iCa concentration, but when 1α was not included in the diet, iCa concentration increased as dietary Ca inclusion level increased (Ca inclusion: *p* = 0.003; Figure 3A). D_3_ + 1α broilers had higher blood bicarbonate concentration (*p* = 0.041), and a trend was observed with bicarbonate concentration with increasing Ca inclusion levels (*p* = 0.08; Figure 3B). Base excess of extracellular fluid (BEecf) is when hydrogen ions diffuse into red blood cells which causes plasma alkalinity to rise as a result as a base excess (25). The statistical model for BEecf initially exhibited a statistical trend with both vitamin D and Ca inclusion. However, the model expresses a statistical difference when only vitamin D is in the model, which is reported in this study. BEecf concentration was increased in D_3_ + 1α broilers (*p* = 0.03; Figure 3C).

#### Plasma Vitamin D_3_ Metabolite Concentration Is Affected by 1α Supplementation and Calcium Inclusion

**Figure 4:**
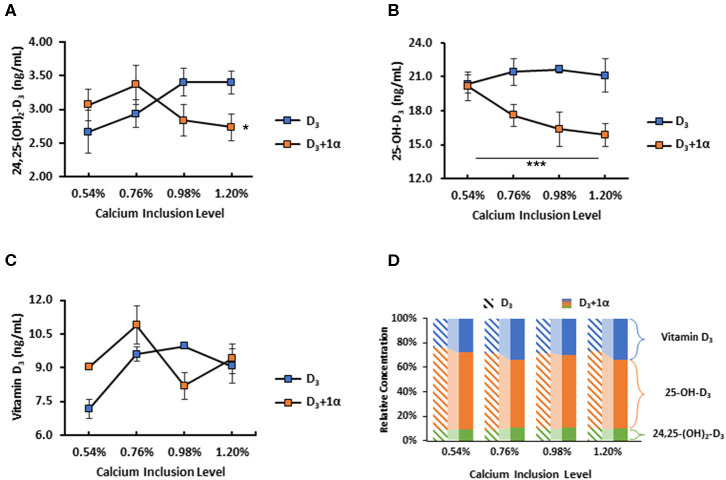
Vitamin D metabolite plasma concentrations of 35 d broiler chickens fed different levels of calcium with or without 1-alpha-hydroxycholecalciferol supplementation (D_3_ + 1α;D_3_, respectively). **(A)** 24,25-dihydroxycholecalciferol (24,25-(OH)_2_-D_3_) **(B)** 25-hydroxycholecalciferol (25-OH-D_3_) **(C)** Cholecalciferol (Vitamin D_3_) **(D)** Comparison of relative concentration between each vitamin D_3_ metabolite between D_3_ and D_3_ + 1α groups; diagonal patterned bars denote D_3_ and solid bars denote D_3_ + 1α. 1α supplementation effect with 25-OH-D_3_. Interaction between calcium inclusion and 1α supplementation with plasma vitamin D_3_. Line graphs show means ± standard error means [*n* = 3; General linear models (GLM), *p < 0.05; ***p ≤ 0.0001].

No difference was noted among dietary treatments with 24,25-(OH)_2_-D_3_ plasma concentration (*p* = 0.19; Figure 4A). Broilers fed 1α supplementation had a decrease in plasma 25-OH-D_3_ concentration (*p* < 0.0001; Figure 4B). There was no dietary Ca inclusion effect on plasma 25-OH-D_3_ concentration. Broilers fed 1α had a linear decrease in plasma D_3_ as calcium inclusion levels increased, except that broilers from 0.76% Ca treatment had the highest D_3_ concentration (*p* = 0.02; Figure 4C). Relative concentrations of each measured vitamin D_3_ metabolite were shown to illustrate how 1α supplementation affected vitamin D_3_ metabolites (Figure 4D).

#### Higher Calcium Inclusion Levels Influenced Duodenal NPTIIb Gene Expression

**Figure 5:**
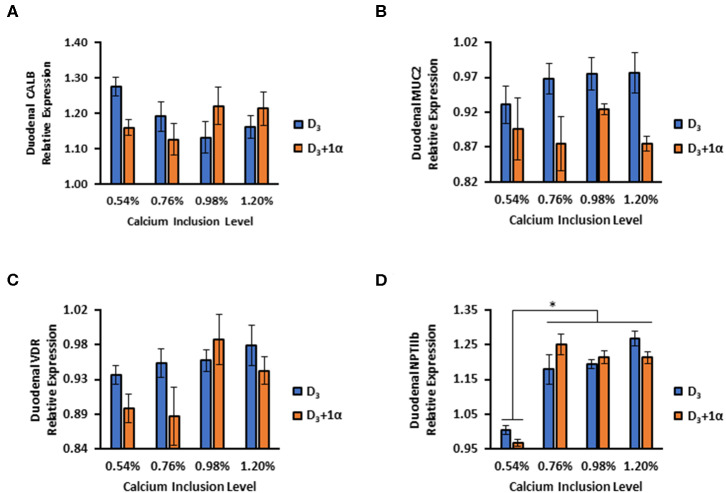
Relative gene expression in duodenal tissue of 35 d broiler chickens fed different levels of calcium with or without 1-alpha-hydroxycholecalciferol supplementation (D_3_ + 1α; D_3_, respectively). **(A)** Calbindin d28k (CALB) **(B)** Mucin 2 (MUC2) **(C)** Vitamin D receptor (VDR) **(D)** Sodium-phosphate cotransporter type II b (NPTIIb). Duodenal tissue was analyzed using qPCR normalized against glyceraldehyde phosphate dehydrogenase (GAPDH; housekeeping gene) expression (*n* = 5). [General linear models (GLM). *p < 0.05].

No Ca inclusion effects or 1α supplementation effects were observed for CALB (*p* = 0.52; Figure 5A). Broilers in D_3_ + 1α treatments had decreased relative expression of MUC2 compared to control treatment (*p* = 0.002; Figure 5B). A statistical trend was denoted for VDR expression for 1α supplementation (*p* = 0.084) and Ca inclusion (*p* = 0.055) with a slight increase in expression with D_3_ broilers, but a larger sample size may be necessary to observe an effect (Figure 5C). D_3_ broilers expressed an increase in NPTIIb expression compared to D_3_ + 1α broilers as dietary Ca increased (*p* = 0.03, Figure 5D).

#### 1α Supplementation and Calcium Inclusion Level Did Not Affect Height/Crypt Depth Ratio of Jejunal Villi

Feeding 1α to broilers had no impact on surface area or height/crypt depth ratio of villi (data not shown; *p* = 0.45 or *p* = 0.86; respectively). Similarly, Ca inclusion levels did not affect either parameter (*p* = 0.40 and *p* = 0.34 for surface area and height/crypt depth ratio, respectively).

## Discussion

Our study indicated that including 1α in young broiler diets can improve their blood iCa status during the starter phase with increased Ca inclusion. 1α supplementation at the grower phase can also cause a decrease in plasma vitamin D metabolites in young broilers. 1α supplementation seems to exhibit greater efficacy during the starter phase compared to the grower phase of broiler diets in its alteration of blood iCa. However, broilers fed grower diets with 1α supplementation had a decrease in plasma 25-OH-D_3_ concentration as Ca inclusion increased. An interesting effect of 1α supplementation on blood chemistry was that it increased BEecf concentration during the grower phase. Increased BEecf concentration indicated broilers were metabolically exceeding their capacity to maintain acid-base balance in body, resulting in more alkali blood, an effect previously described by Mongin (26). We argue that this effect is caused by excess dietary Ca which has electrolytical potential as a cation when present in tissues (27), which has biological implications for potentially using 1α in diets with lower Ca levels for the grower phase; otherwise, these birds are under some form of duress to reduce BEecf to homeostatic balance.

Blood Ca is tightly regulated for various reasons including the need to control Ca distribution into tissues and to maintain blood pH (28). Levels of iCa in our two experiments were similar in range to those studies which reported plasma Ca concentrations from young broilers of similar ages (21, 29, 30), indicating no unusual physiological levels to denote Ca deficiency or toxicity. For broilers from the starter diet experiment, at 1.10% Ca inclusion, D_3_ + 1α broilers had decreased iCa compared to D_3_ broilers; this exhibits 1α's increased efficacy on Ca utilization because D_3_ + 1α broilers most likely absorbed Ca for bone metabolism compared to D_3_ broilers having a much higher blood iCa concentration. Han et al. (21) fed broiler chicks different levels of 1α and reported a linear effect with increasing 1α also increased plasma Ca concentration, although their broilers had a higher average concentration compared to our birds by almost 1 mmol/L with their 5 μg/kg 1α supplementation with 0.25% Ca diet. However, their plasma Ca was collected at 21 days compared to our study, which collected iCa at 15 days. Growth performance of these birds had a quadratic relationship with Ca inclusion and increased Ca digestibility with chicks fed 1α relative to Ca inclusion levels (23). Further investigation with an even lower dietary Ca inclusion level may help fit a better regression line for this relationship with 1α's efficacy on Ca absorption into bone. Examining hydroxylase expression in kidney and liver tissues could determine whether pathological issues are developing due to Ca intake. There would be greater expression of 25-hydroxylase in liver with regard to 1.40% Ca inclusion level of D_3_ + 1α broilers having a higher iCa concentration compared to their counterparts without 1α supplementation. If this case were to occur, then histopathological examination of kidneys and liver would verify soft-tissue calcification.

When broilers were fed at the grower phase, increasing Ca inclusion levels caused an increase in blood Ca concentration at the grower phase, regardless of 1α supplementation. Also, both Ca inclusion levels and 1α did not affect body weight of broilers fed at the grower phase (23). Our result is in agreement with that of Sebastian et al. (31) who also report how increasing Ca inclusion levels led to increased blood Ca concentration. Increased blood Ca concentration indicates that Ca requirements are met in these chickens and that Ca is most likely being excreted or stored in bone, which was observed with various strains of broilers because of how tightly regulated blood Ca is (29, 31). It should also be noted that 1α caused birds to have a consistent Ca digestibility, whereas birds not fed 1α had a negative quadratic relationship with Ca digestibility as Ca inclusion increased (23). 1α's impact on increasing blood bicarbonate concentration is probably caused by a shift in acid–base balance. We did not observe any changes in pH or CO_2_ blood concentration; however, we speculate bicarbonate's increase without any change to CO_2_ implies these broilers may be under physiological stress with alkalosis. Metabolic alkalosis can be caused by chloride depletion but can also be caused by hypercalcemia because hypercalcemia increases bicarbonate resorption (32). Therefore, it is possible that 1α, with addition of dietary vitamin D, could have caused greater Ca absorption that led to greater bicarbonate being resorbed. There is also the possibility that at the time of blood sampling, the broilers may have metabolically compensated for the partial pressure of CO_2_ to maintain pH in the reaction to increased bicarbonate levels (33). A caveat of our study is that we did not examine blood chloride levels, which could potentially explain the BEecf increase. Future studies should explore the mechanism with Ca inclusion in broilers on why blood bicarbonate and BEecf increased even though pH did not change.

There were no differences in vitamin D_3_ metabolite blood concentrations in broiler chicks fed starter diets with or without 1α supplementation at 0.95% Ca inclusion. 1α may not be affecting vitamin D pathways considering there was no difference in blood iCa concentration at this level. Based on data from the National Research Council (34), 0.95% Ca inclusion is closest to the starter phase minimum requirements for broilers. We may find a difference in vitamin D metabolite blood concentrations in broilers fed 1.10% Ca because of how drastically iCa concentration differed between D_3_ and D_3_ + α broilers.

However, 1α's effects are more pronounced when broilers are older and fed a grower diet. An inverse relationship of vitamin D_3_ plasma concentration implies how 1α's influence in vitamin D metabolism is dependent on dietary Ca inclusion (Figure 4C). This relationship is also connected to 25-OH-D_3_ because 1α is likely exerting an effect in order to reduce vitamin D_3_ because of 1α's efficacy. We believe D_3_ is being excreted in feces, but we are unable to find any poultry-related studies that report vitamin D in excreta. A human study discussed how 25-OH-D_3_ was intravenously administered to patients and positively correlated to increased 25-OH-D_3_ in urine (35). It is important to note that broilers grown to the grower phase for this study were fed common starter diets with 1α supplementation. When these broilers were sampled, broilers not fed 1α in the grower phase had a period of 18 days since they were given 1α supplementation. However, this may not impact our findings because in plasma, 1,25-(OH)_2_-D_3_ has a half-life of about 10–20 h and 25-OH-D_3_ is about 15 days (14). Considering broilers not fed 1α had consistent plasma levels of 25-OH-D_3_, it is unlikely that the 1α they were fed from the starter diet was remaining in their bodies because it would have been converted to 1,25-(OH)_2_-D_3_ and excreted or used up before blood was collected.

**Figure 6:**
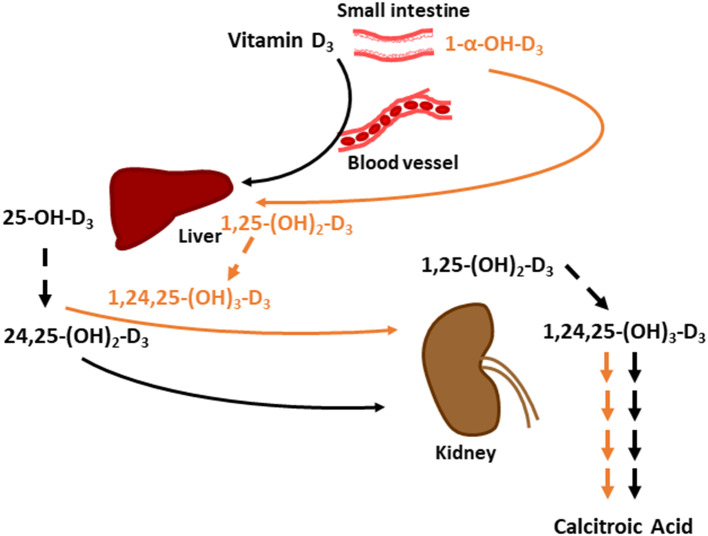
Hypothetical model on comparing how dietary vitamin D_3_ and 1-α-hydroxycholecalciferol (1α) are converted to water soluble calcitroic acid to be excreted. Black arrows denote vitamin D_3_'s pathway to calcitroic acid and orange arrows denote 1α 's pathway. Dashed arrows signify 24-hydroxylation step. Multiple arrows between 1,24,25-(OH)_3_-D_3_and calcitroic acid denote number of conversion steps. 25-OH-D_3_ is 25-hydroxycholecalciferol; 24,25-(OH)_3_-D_3_ is 24,25-dihydroxycholecalciferol; 1,25-(OH)_3_-D_3_ is 1,25-dihydroxycholecalciferol; and1,24,25-(OH)_3_-D_3_ is 1,24,25-trihydroxycholecalciferol.

Excess 25-OH-D_3_ in chickens is converted to 24,25-(OH)_2_-D_3_ by 24-hydroxylase in kidneys (36). 24,25-(OH)_2_-D_3_ is an inactive vitamin D form that is excreted, but this form is not the end-product of this pathway; 24,25-(OH)_2_-D_3_ goes through a series of conversions and ends up becoming calcitroic acid, a water-soluble molecule that is readily excreted (37). Although no difference in 24,25-(OH)_2_-D_3_ plasma concentration was observed, we speculate that the calcitroic acid concentration would be higher in 1α-fed broilers because 1α cannot be converted to 24,25-(OH)_2_-D_3_. 1α would likely be converted to 1,25-(OH)_2_-D_3_ and then undergo multiple conversions to become calcitroic acid. We constructed a hypothetical model to compare how dietary D_3_ and 1α would be converted to calcitroic acid to be excreted (Figure 6). 1α-fed broilers exhibiting a linear drop in 25-OH-D_3_ plasma concentration are a consequence of 1α being converted to 1,25-(OH)_2_-D_3_, which will reduce the amount of vitamin D_3_ being converted into 25-OH-D_3_ due to a negative feedback loop (38). As Ca inclusion increased in 1α-fed broilers, 25-OH-D_3_ concentration likely dropped because of 1α's effects on Ca absorption. Purifying fecal content to measure vitamin D may provide insights to how much D_3_ was excreted relative to how much was fed.

For broilers sampled during the grower phase, broilers fed 0.54% Ca without 1α had about 0.18 relative expression increase of CALB compared to control (D_3_, 0.76% Ca inclusion): this observation could signify that broilers were trying to bind dietary Ca for transport and absorption. Our results were similar to a different study by Li et al. (39) in which low Ca upregulated CALB expression. Although our results indicated MUC2 expression was statistically lower in D_3_ + 1α broilers, relative expression was only decreased by about 0.5 compared to D_3_-fed broilers, which indicates no change in MUC2 expression. This finding is indicative that these broilers can be considered healthy, an inference supported by the FITC-D data, which denoted no difference in jejunal morphology.

VDR is responsible for signal transduction of Ca absorption genes when 1,25-(OH)_2_-D_3_ binds to it (40). For VDR expression in duodenum of 35-day old broilers, even though it was not statistically different, a trending increase of VDR expression as Ca inclusion increased for broilers not fed 1α indicates dietary Ca's regulatory role with increasing VDR expression. VDR's role as a signal transducer for gene expression for proteins (TRPV6 and CALB) explains why vitamin D is important for dietary Ca absorption (41).

**Figure 7:**
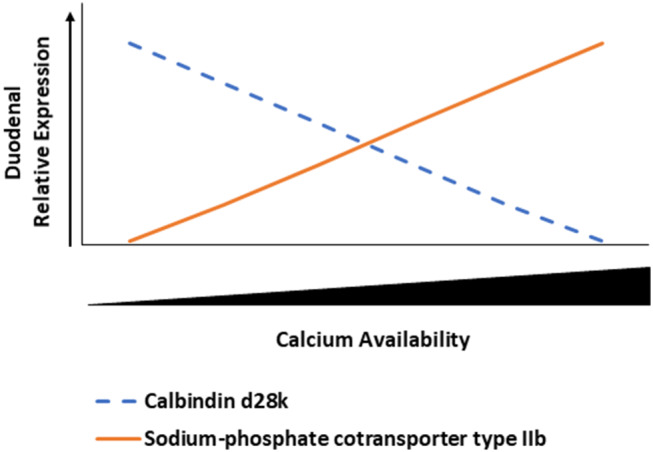
Inverse relationship between duodenal calbindin d28k (CALB) and sodium-phosphate cotransporter type IIb (NPTIIb) in broiler chickens relative to calcium availability. As duodenal calcium concentration increases, then CALB expression decreases and NPTIIb expression increases to potentially maximize phosphate absorption because of the potential excessive calcium binding to phosphorus to form tricalcium phosphate and making phosphorus unavailable.

NPTIIb is an important cotransporter protein that requires sodium to move P in its phosphate form into cells from the intestinal lumen (42). Increasing Ca inclusion led to an increase in duodenal NPTIIb expression in 35-day D_3_ broilers, which was likely caused by P imbalance. Even though our study did not include citric acid, there was a study that noted how 1α, phytase, and citric acid increased phytate *P* utilization in broiler chicks (22). Excess dietary Ca compared to *P* can cause Ca to bind to *P* and form insoluble tricalcium phosphate (43). Our results were similar to Li et al. (39), in which increased dietary Ca levels led to increased NPTIIb expression in duodenum. Li et al. also noted how high dietary Ca could cause a decrease in available *P*, which triggers the increased expression of NPTIIb in the duodenum. A proposed model of the inverse relationship between CALB and NPTIIb expression relative to Ca inclusion levels is denoted for broilers not fed 1α (Figure 7). Further characterization of this relationship can elucidate the need to reevaluate nutrient interrelationships in animal production. There may be unintended impacts of using excessive nutrients as a safety net for meeting nutrient requirements because potential deficiencies can be caused by nutrient antagonism, but these consequences can be detected by examining genes related to absorption such as NPTIIb and CALB.

Our work highlights that 1α supplementation with certain levels of Ca inclusion can impact blood Ca concentration and affect vitamin D metabolite concentration. Our findings exhibit that 1α can improve Ca utilization in young broilers with an implication for reducing dietary Ca in their diets. We suggest 1α should be supplemented in broiler diets for the starter phase and either removed for the grower phase or provided with reduced dietary Ca levels. 1α's potential to reduce dietary Ca without any negative impacts on growth performance signifies the importance of synthetic nutrients for improving animal production while reducing potential environmental impacts from excreted excess nutrients. Future research should explore how much 1α supplementation will cause vitamin D toxicity in growing animals to characterize an animal's regulatory limits of removing vitamin D with consideration of 1α's bypassing of negative feedback regulation.

## Data Availability Statement

The raw data supporting the conclusions of this article will be made available by the authors, without undue reservation, to any qualified researcher.

## Ethics Statement

The animal study was reviewed and approved by Institutional Animal Care and Use Committee, North Carolina State University.

## Author Contributions

MW, JF, and KL contributed to the conception and design of the study. MW and KL contributed to the acquisition, analysis, interpretation of data, wrote the first draft of the manuscript, and were responsible for the final editing of the manuscript. MW, TV, OT, and KL contributed to the manuscript. All authors read and approved the final manuscript.

## Conflict of Interest

The authors declare that this study received funding from Premex. The funder had the following involvement with the study: conception and design of the study.
